# What Are the Best Methods for Obtunding Sensibility of the Dentine by Either Local or General Means? Should Arsenic Ever Be Used?

**Published:** 1894-04

**Authors:** Wm. H. Trueman

**Affiliations:** Philadelphia.


ARTICLE IV.

### WHAT ARE THE BEST METHODS FOR OBTUNDING SENSIBILITY OF THE DENTINE BY EITHER LOCAL OR GENERAL MEANS? SHOULD ARSENIC EVER BE USED?

BY WM. H. TRUEMAN, D. D. S., PHILADELPHIA.

Read before the Pennsylvania Association of Dental Surgeons.

To fully answer this question a volume might profitably be written. Indeed, were this audience students who were as yet at the threshold of dental science, justice could not be done to it in so brief an essay as the present occasion calls for. Recognizing. however, that the attempt is to simply promote and facilitate an orderly interchange of mutual experiences, I shall, as it were, merely catalogue the more prominent features, presuming while so doing that on this subject we are all, in theory and practice, equally well posted.

I take it that by sensitive dentine we understand an *abnormal* sensitiveness of this portion of tooth tissue. We recognize that while cutting into tooth tissue, either normal or carious, that there is to be expected an appreciable degree of discomfort or pain; indeed, in practice, the absence of sensation invariably suggests a doubt of the tooth's vitality. In a large majority of cases this discomfort is quite bearable; so slight, indeed, that we would hardly call it pain. We recognize, also, that this discomfort may be greater or less as the cutting tool encroaches upon or is remote from those portions of the tooth that normally are, as we term it, sensitive. It is only when this discomfort becomes excessive, when it unduly taxes the endurance of the patient, that we speak of it as sensitive dentine, and seek for some means to obtund it. In its origin the cause of sensitive dentine may be systematic or local. This must be considered when seeking means to control it. So much, indeed, does the choice of means depend upon the cause that I cannot see how they can profitably be separately considered. In answering the question I shall take the liberty of substituting the word "controlling" in place of "obtunding"; the words are not in this connection synonyms; "controlling" has a broader meaning, and its use permits a more practical discussion of the subject. The desired object in many cases is best attained and the sensitiveness controlled by managing the patient, rather than by applications to the sensitive portions of the tooth.

We will first consider systematic causes and systematic treatment; not, perhaps, in that order a careful study of the subject would suggest, but rather impromptu.

We recognize, first, that either from their physical make-up or the result of mal-education some patients feel pain more acutely or bear it with less fortitude than do others. How to differentiate these two conditions and the best means of treatment must be learned from practical experience: it can be acquired in no other way.

In the last number of the *International Dental Journal* (May, 1893), are two papers that I have read with unusual interest. One is by Dr. Hamilton Osgood, of Boston, Mass., upon "The Use of Hypnotism in Medicine;" the other by Dr. Thomas Fillebrown, also of Boston, upon "The Use of Hypnotism in Dentistry." Both are unsatisfactory in that, not having been originally written for publication, but reported papers read at a dental gathering, a clear and explicit understanding of them cannot be had without the illustrative demonstration accompanying the reading, at least by those so little acquainted with the subject that their imaginations fail to supply the deficiencies. I have always been impressed that hypnotism, mesmerism, animal magnetism, etc., were so closely allied to empiricism as to be unworthy of notice. If the claims made for it by these gentlemen, both of whom are entitled to respectful consideration, should prove to be tenable, it may perhaps be useful in treating such cases as these. Indeed, it is a question whether or not we have been unwittingly using it. Just what the gentlemen mean by hypnotism, or how they bring the patient so completely under their control, I cannot quite grasp. It seems, however, very like a recital of that which no doubt is to each one of us a daily experience. The restless patients we control at times by commands, by persuasion, or by assurance. So far as I can gather, this hypnotism is produced by assuring the patient that there will be no pain, that there is no pain, that it is not hurting, and thus by progressive assurance and constantly insisting overrule the patients' will so completely that they are not conscious of suffering, and are, as it were, asleep. There is much in this; by judicious suggestion we can very often so quiet restless patients as to facilitate our work. I have never, however, been able to control them to that extent claimed by Drs. Osgood and Fillebrown. My patients have an annoying habit of waking up too soon and at inconvenient times. When we remember how mysterious are the workings of the human mind, it is not best to hastily dismiss anything of this kind that promises to be of practical usefulness simply because it does not conform to our ideas of that which we are pleased to call "science." A few years ago I met a gentleman of education and intelligence who had been bedfast for months from rheumatism, scientific medicine having failed to give him relief. At the suggestion of a friend he sent to an advertising quack for a magnetic ring, a ring made of zinc, into which were inserted a few rivets of copper, and intended to be worn on the finger. A week after putting it on he was out, and in a few weeks more, free from pain and stiffness, was attending to his business a well man.

Another case: A gentleman I well know was bedfast and helpless, I think from a rheumatic affection, and had been so for some time with but little hope for recovery. One night while his attendant was absent, the lamp (one using the old-fashioned burning fluid in use some forty years ago, a mixture of alcohol and spirits of turpentine I think it was) exploded, setting fire to the room. His cries for help were not heard; the fire reached the bed; in his excitement he arose and worked so vigorously in extinguishing the fire that when help arrived the danger was over. The man, who a few moments before was so helplessly ill, did not return to bed; he dressed, and the next day was up and about, superintending his business. During the excitement his disease left him and did not return. He lived in the enjoyment of good health some thirty years, dying of old age an octogenarian. And still another case: A dentist, a graduate of the old Philadelphia College in 1855 or 6, told me a few years ago that while suffering from a distressing malady that his medical attendant had failed to relieve, he was advised to carry in his pantaloons pocket of the afflicted side a round potato about the size of a walnut. Having but little faith in it, and feeling not a little ashamed of the weakness it indicated, he quietly pocketed the potato, and within a short time felt decidedly relieved, so much so that he at once stopped taking medicine and soon regained his usual health. The experience he had several times repeated on a recurrence of the trouble with a like satisfactory result.

And still another case: A maiden lady of means, refinement, and education, aged about fifty, very deaf, so much so that she carried with her at all times a tablet and pencil, which she handed to those with whom she desired to communicate, with the request, "Will thee kindly write, I cannot hear," while visiting a religious meeting at which the efficacy of prayer was being considered, she was strongly impressed. She arose, stated her affliction and how seriously it impaired her usefulness in the charity work to which her life was devoted, and asked for the prayers of the audience that her hearing might be restored. Her remarks were so earnest and so fully in accord with the feeling pervading the meeting that the request was at once acceded to. Before the meeting closed she announced that the prayers were answered, and amid much religious excitement declared that she conld hear perfectly. I saw her a few months after. She grasped me warmly by the hand and with much feeling exclaimed: "Doctor, I shall not trouble thee again to write on the tablet; my hearing has been restored; I can hear perfectly;" and then she related her experience. I soon found, however, that this was a delusion. Although she declared that it was not at all necessary that I should raise my voice, she could hear a whisper, I found it utterly impossible to communicate with her without reducing to writing all I had to say. Before, I could, by speaking very loud and distinct, and occasionally repeating, carry on a conversation after by writing our minds has been brought into accord; but now, while she answered questions and replied to remarks promptly, her answers and replies were not to what she really heard, but to what she thought had been said, and were appropriate only by mere accident. The pencil and slate were more than ever necessary; yet, strange to say, *that* she did not seem to appreciate, and while accepting the assistance acted as though I used them from force of habit only. Her relatives and associates tell me that they find increased difficulty in communicating with her from the fact that she does not seem to appreciate her deafness. A year later, when last I saw her, she was still under the delusion. She is so much brighter, more cheerful, and seems in every way so happy in the delusion that her friends make no effort to combat it. In every other respect she is mentally and physically unchanged. Now, is this a phase of hypnotism? Could we make our patients, while otherwise unchanged, as oblivious to pain as she is to her deafness, what a boon it would be.

These are but types of cases that, well authenticated, can be duplicated indefinitely. We may laugh at these things, but that neither refutes nor explains them. They are but illustrations of the thousand and one ways in which the thing we call "mind" controls the other thing we call "matter." The patient who feels that liniments and doses scientifically prescribed and faithfully used were being used in vain, have the best of us when they say, "I put on the ring and was soon well," "I pocketed the potato and felt like a new man." They wanted to be rid of their ailment; this accomplished, whether it was a mere coincidence or a result is to them a small matter. If hypnotism, or mesmerism, will enable us to work upon sensitive teeth with comfort to ourselves and our patients —that is the practical point—let us use it and study the science of it at our leisure. I am not as yet a convert; I confess, however, to being a little drawn towards the anxious bench. It is worth looking into.

With some women patients there are times, as we all recognize, when to use a well understood expression, "their nerves are high strung," and dental operations are more than usually trying. This many of our patients have learned from experience, and I am frequently conscious that it is considered when making appointments. The remark, "If it will make no difference, I would rather defer it a week or two; I will then be better able to bear it," usually refers to it. At other times the dressmaker is a convenient excuse for a prudent postponement. Among many valued suggestions of my preceptor was that when the teeth of women patients seem more than usually sensitive a postponment of painful operations will always be in order. A week or ten days delay has in a multitude of cases been of more practical value than any medication that could have been used.

A successful physician's arcanum, and a successful dentist's also, I take it, will ever be in placing "tact" before "physic." Dismissing new cases calling especially for tact,

I will now ask your attention briefly (there is in it but little that is new) to cases calling for physic.

Active systematic treatment (dosing) to reduce hyper-sensitiveness of dentine should be cautiously approached. The use of sedatives, such as bromides, has been suggested, and in a limited number of cases it has seemed to have value, but there is always a risk, and I have not thought that, except in unusual cases occurring at long intervals, a dentist is justified in their administration. Their general use would undoubtedly be productive of much mischief. No matter how thorough has been his education, how well posted, or how scientific a dentist he may be, if he have the necessary practice to acquire and maintain a fair degree of skill as a dentist, he has neither time or opportunity to become skilled in the use of medicine. It is not book-learning, it is not attending a medical course, but it is the practical knowledge which comes from every-day use of medicine and constant observation of their effect under varied circumstances, that gives the experience, without which the adminstration of medicine is an *unjustifiable tampering with a patient's health.* In my judgment, a wise dentist in treating these cases will confine himself closely to local applications.

For the present purposes we will consider local cases calling especially for local treatment, to refer to those cases where the hyper-sensitiveness is localized, as for instance in cervical cavities, this distinction, be it understood, is merely for convenience. First in importance and the most reliable and generally useful is dryness of the cavity, and next to this we may name heat; they are, however, closely associated. Since Professor Thomas C. Stellwagen, of this city, many years ago suggested the glowing end of a charred match stem, a legion of new agents for, and new methods of, applying heat have appeared. I question, however, if in any of them there is any virtue apart from the greater or less dryness resulting. So far as I have experimented I have not been impressed that the various methods have any practical advantage, the one over the other, apart from the greater or less convenience of their application. There is this that I have found, and I presume it is a common experience, that all new methods for obtunding sensitive dentine at first work like a charm, they are the very things we have long waited for, yet one after the other in a short time are laid aside. Phosphoric acid, the essential oils, and a multitude of obtunding agents have had their day. Silver nitrate, so useful in rendering less sensitive exposed tooth tissues, has with me proved of little value in preparing cavities. Zinc chloride holds its own, but I seldom use it on account of the acute pain attending its application. Robinson's remedy has proved, perhaps, as generally useful as anything that I have tried. The application of cold, if so unscientific an expression may be permitted, has from time to time had its advocates. My experience with rhigolene and ether spray in 1866 impressed me unfavorably. I have seen nothing in later freezing methods to warrant a re-trial.

Now, in conclusion. Shall arsenic ever be used? An appropriate answer to this question may be found in Dr Elisha Townsend's address before the American Association of Dental Surgeons, at the meeting in Baltimore, March, 1850, in which he recommends that the amalgam pledge adopted by that society in 1845 be rescinded. (See *Dental News Letter*, Vol. 4, page 65.) His objection that the amalgam pledge was "an embargo upon opinion' applies with equal force to any action intended to restrict individual judgment in the use of arsenic. Any dentist who is as well informed as every dentist should be regarding the immediate and ultimate action of arsenic so used is quite competent to decide whether the best interests of the patient will or will not be served by its use. During my professional life I have three times used it to obtund sensitive dentine, and would not hesitate to do so again should a case present where I thought it the best thing to do. One case has passed out of my mind; the other two patients I have continuously waited upon, and have seen within a few weeks. Allow me to relate briefly their history.

In 1871, a lady aged about nineteen presented. She had been ill a long time, said to be from a spinal trouble. During the preceding few years all attempts to operate upon her teeth had failed. The teeth were excessively sensitive; the patient from continuous suffering was unable to bear the necessary manipulation; and imperfect work had been the result. The teeth had become more and more sensitive, and so aggravated the systemic conditions that the point had been reached when something *must* be done. Her physician declared that until the dental irritation had been allayed he could do nothing, and in a message to me he said she could not much longer bear her suffering, and that unless the teeth could speedily be made comfortable he advised their extraction, but doubted seriously if she could survive the shock. Desperate cases demand desperate remedies. Selecting two cavities that seemed to be giving rather more trouble than the others, although they were comparatively superficial, I applied arsenic, allowing it to remain about eight hours. Immediately upon its removal, the cavities, now but slightly sensitive, were excavated, the cutting being continued until sensitive tissue was encountered, and gold fillings were inserted. These fillings I saw last February, and after twenty-two years service they are still in good order. Of course both pulps died; that I expected when the application of arsenic was made. The treatment of the roots, however, was deferred until the patient's health had materially improved. The first to show signs of devitalization was opened into some nine months after; the other gave no evidence of loss of vitality until after the lapse of two years. After the dental irritation had been allayed the improvement in the general health was immediate and gratifying. The less serious cases were then taken in hand at long intervals; from the beginning of March to the end of November fourteen fillings were inserted, and the teeth then pronounced in good order, the general health being nearly restored. I am impressed that in her case the arsenical application was the turning point. She told me then, and has often repeated it, that the night after the fillings were inserted was the first restful night's sleep she had had for months.

The other case was treated during May, 1869. A Miss, aged about thirteen, in poor health, from causes incident to her age and sex. Two superficial cavities upon the anterior approximal surfaces of the superior central incisors were very sensitive, and evidently aggravating the systematic conditions. After repeated failures to so obtund the sensitiveness that the cavities could be filled, arsenic was used as a last resort and with excellent effect. The application was allowed to remain over night, the cavities immediately excavated until sensitive tissue was reached, and filled. These were closely watched. November, 1873, one was found to be devitalized and the root treated. The filling in that tooth was renewed in 1882. To the other tooth nothing has been done; the filling inserted in 1869 still remains. I saw it a few weeks ago; I *presume* that it is devitalized, but, not being sure, prefer to let it alone.

Whether with present means to treat such cases I would again use arsenic I cannot say. They were exceptional cases that occur at long intervals. I do not at this time recall any precisely like them.

I had a lesson in treating sensitive dentine, while attending lectures in 1864, from which I have profited much. I was operating upon a young patient, whose restless movements greatly embarrassed me. Professor C. N. Peirce, then lecturer upon operative dentistry, passing through the clinic room, attracted no doubt by my patient's outcries, paused for a few minutes at the chair. In a quiet tone he spoke a few kind, encouraging words, "Bear it patiently—it will soon be over—a little more courage,"

etc. The effect was magical; was it hypnotism? The patient settled back in the chair and remained perfectly still. In a few minutes the cavity was prepared and the trouble over.

I study my patients and endeavor to learn their peculiarities and how best their attention can be attracted and held from the work in hand. This and sharp, judiciously used instruments have with me to a great extent solved the problem of controlling sensitive dentine. It is seldom, indeed, that I have recourse to an obtunding agent other than the dryness secured by the use of the rubber dam. Notwithstanding this, I am not willing to say that arsenic should never be used; I would prefer to say "hardly ever." —*Office and Laboratory.*

